# Study on the Mechanism of Capillary Leakage Caused by Hypoxia-Inducible Factor-1*α* through Inducing High Expression of Matrix Metalloproteinase-9

**DOI:** 10.1155/2021/9130650

**Published:** 2021-09-16

**Authors:** Huili Li, He Huang, Yunliang Cui, Weiwei Li, Shuliu Zhang, Yugang Chen

**Affiliations:** Department of Critical Care Medicine, The 960th Hospital of the PLA Joint Logistics Support Force, Jinan 250031, Shandong, China

## Abstract

**Purposes:**

This study mainly explored the mechanism of capillary leakage caused by hypoxia-inducible factor-1*α* through inducing high expression of matrix metalloproteinase-9. *Method. *We established a monolayer endothelial cell model by culturing human umbilical vein endothelial cells (HUVEC) in vitro, used tumor necrosis factor (TNF*α*) and HIF-1*α* inhibitor 2-methoxyestradiol (2ME2) to act on HUVEC, and at the same time constructed siRNA-transfected HUVEC to interfere with the expression of HIF-1*α*. The permeability of monolayer endothelial cells was measured by transwell chamber method, the concentration of MMP-9 in the supernatant was measured by ELISA method, the expression of key molecules related to permeability (HIF- 1*α*, MMP-9, claudin-5, and ZO-1) was measured by RT-PCR and Western blot method, and the localization and expression of claudin-5 and ZO-1 were measured by immunofluorescence method. We searched for 7 HIF-1*α* hypoxia response elements within 4000 bp before the transcription start site in the MMP-9 promoter region, constructed the MMP-9 promoter-luciferase reporter gene recombinant plasmid, transfected and stimulated HUVEC with TNF*α*, and detected the effect of 7 hypoxia response element plasmids on the transcription activity of MMP-9 promoter.

**Results:**

Under the action of TNF*α*, the permeability of monolayer endothelial cells increased, and the concentration of MMP-9 in the cell supernatant increased. 2ME2 and HIF-1*α*-siRNA transfection can improve the above situation (*P* < 0.05). 2ME2 and HIF-1*α*-siRNA transfection can inhibit the high expression of HIF-1*α* and MMP-9 caused by TNF*α*, thereby increasing the expression of claudin-5 and ZO-1 (*P* < 0.05). 2ME2 and HIF-1*α*-siRNA transfection can reduce the inhibition of TNF*α* on the expression of cell membrane protein claudin-5 and tight junction protein ZO-1. Element 1, element 5, and element 7 are the sites where HIF-1*α* interacts with MMP-9 at the transcription level.

**Conclusion:**

This study shows that HIF-1*α* can increase the permeability of monolayer epithelial cells by inducing the high expression of MMP-9, leading to capillary leakage. Its target is at the −3798 bp, −1878 bp, and −1489 bp points of the transcription initiation site in the MMP-9 promoter region.

## 1. Introduction

Vascular leakage is the main reaction of tissue damage [1]. The destruction of the endothelial barrier and vascular leakage are characteristics of a variety of life-threatening diseases, including sepsis, acute respiratory distress syndrome (ARDS), and COVID-19, which have an important impact on the morbidity and mortality of critically ill patients [2–4]. Despite the serious adverse clinical results associated with vascular leakage, there is currently no therapy to reverse the destruction of the endothelial barrier [5]. Therefore, it is necessary to study the underlying mechanism of vascular leakage.

HIF-1 is a heterodimer composed of *α* subunit and *β* subunit. HIF-1*α* (hypoxia-inducible factor-1a) encodes the *α* subunit of the transcription factor hypoxia-inducible factor-1 (HIF-1). HIF-1 activates the transcription of many genes, including genes involved in energy metabolism, angiogenesis, and apoptosis and genes generating other protein products that can increase oxygen transfer or promote metabolism to adapt to hypoxia, as a main regulator in the homeostatic response to cells and the whole body hypoxia. HIF-1 plays an important role in embryonic angiogenesis, tumor angiogenesis, and pathophysiology of ischemic diseases. For example, it has been reported that HIF-1*α* promotes tumor chemotherapy resistance by recruiting GDF15-induced tumor-associated macrophages in colorectal cancer [6]; silencing long noncoding RNA NEAT1 stimulates HIF-1*α*/NF-kappaB by competitively binding miR-33a-5p to inhibit the occurrence of infantile hemangioma [7]; under hypoxic conditions, miR-375 impairs the invasion ability of liver cancer cells through targeted regulation of HIF-1*α* [8]; in the process of IL-1*β*-induced chondrocyte degeneration, FBW7 regulates the HIF-1*α*/VEGF pathway [9]; miR-210 regulates the inflammatory response of exudative otitis media by inhibiting the expression of HIF-1*α* [10]; extracellular vesicles from healthy cells activate and improve the cell function of progeria stem cells and stem cells themselves through miR-302b and HIF-1*α* [11].

Proteins of the matrix metalloproteinase (MMP) family are involved in the degradation of extracellular matrix in normal physiological processes, such as embryonic development, reproduction and tissue remodeling, and disease processes such as arthritis and metastasis. Most matrix metalloproteinases are secreted in the form of inactive proproteins and are activated when cleaved by extracellular proteases. MMP9 (matrix metallopeptidase 9), as a member of the matrix metallopeptidase family, also plays an important role in many diseases and life processes. In dentistry, MP-8, MMP-9, and BOP can be used to assess the periodontal condition of orthodontic patients [12]; in otology, the functional polymorphism of MMP9 and BDNF enables them to be used as potential biomarkers of neuroplasticity in the cochlear implantation treatment for the prelinguistic deaf [13]. Active MMP-9 increases at 7 and 14 days after kidney transplantation and returns to baseline level 3 months after kidney transplantation, consistent with the improvement of renal function and plasma creatinine. In dialysis patients and kidney transplant recipients, active MMP-9 is associated with pulse pressure as an indicator of arteriosclerosis. Therefore, active MMP-9 can be used as a biomarker of arterial stiffness in renal replacement therapy [14]; in patients with colorectal liver metastasis, high expression of MMP9 indicates that patients can get a better prognosis [15]; in addition, excessive activation of MMP-9 will hinder the wound healing of diabetic skin. In diabetic skin tissues, TET2 can induce demethylation of the MMP-9 promoter, leading to high expression of MMP9 [16]; increased expression levels of MMP2 and MMP9 in the premature myometrium will aggravate uterine contractions [17]; MMP9 can also regulate the integrity of blood-testis barrier to prevent virus invasion and protect the body [18]; inhibiting MMP9 can weaken migration, invasion, and colony formation and promote CD8(+) T cell infiltration and activation. Interestingly, the primary tumor was not affected, which indicates that inhibiting active MMP9 is mainly effective in the early metastatic cascade.

However, there are still very few studies on the mechanism of HIF-1*α* on vascular leakage. Therefore, this study explored the mechanism of HIF-1*α* inducing the high expression of MMP-9 and causing capillary leakage.

In this study, a monolayer endothelial cell model was established by culturing human umbilical vein endothelial cells (HUVEC) *in vitro*, using tumor necrosis factor (TNF*α*) and HIF-1*α* inhibitor 2-methoxyestradiol (2ME2) to act on HUVEC, and constructing siRNA-transfected HUVEC to interfere with the expression of HIF-1*α*. The permeability of monolayer endothelial cells was measured by transwell chamber method, the concentration of MMP-9 in the supernatant was measured by ELISA method, and the expression of key molecules (HIF-1*α*, MMP-9, claudin-5, and ZO-1) related to permeability was measured by RT-PCR and Western blot method. The localization of claudin-5 and ZO-1 was observed and the protein expression was reconfirmed by the immunofluorescence method. The MMP-9 promoter-luciferase reporter gene recombinant plasmid was constructed and transfected, TNF*α* was used to stimulate HUVEC, and the effect of 7 hypoxia response element plasmids on the transcription activity of MMP-9 promoter was detected.

## 2. Materials and Methods

### 2.1. Cell Culture and Cell Transfection

HUVEC cells were cultured in DMEM high glucose medium (containing 10% fetal bovine serum) and placed in an incubator (37°C, 5% CO2), and the cell culture medium was replaced every 2 days until it grows and converges to 70–80% passage. 3–4 times passage was taken for experiment and divided into 7 groups, namely, the normal control group, only adding DMEM high glucose medium containing 10% fetal bovine serum; TNF*α* group, adding DMEM high glucose medium containing 50 ng/ml TNF*α*; TNF*α* + 2ME2 (0.1 uM) group, adding DMEM high glucose medium containing 50 ng/ml TNF*α* and 0.1 uM 2ME2; TNF*α* + 2ME2 (0.01 uM) group, adding DMEM high glucose medium containing 50 ng/ml TNF*α* and 0.01 uM 2ME2; TNF*α* + 2ME2 (0.001 uM) group, adding DMEM high glucose medium containing 50 ng/ml TNF*α* and 0.001 uM 2ME2; HIF-1*α* siRNA-negative control group, negative HIF-1*α* siRNA was used to transfect HUVEC, and after monolayer cells were formed, 50 ng/ml TNF*α* was added to act for 24 hours; HIF-1*α* siRNA-positive transfection group, positive HIF-1*α* siRNA was used to transfect HUVEC, and after monolayer cells were formed, 50 ng/ml TNF*α* was added to act for 24 hours. This study was approved by the institutional ethical review board of the 960th Hospital of the PLA Joint Logistics Support Force, China.

### 2.2. Transwell Experiment

HUVEC was seeded in the upper chamber of the transwell (1 × 10^5^/well) to establish a complete monolayer endothelial cell model. After drug action as grouped, we washed with PBS for 3 times, seeded 100 *u*l PBS which contains FITC‐Dextran40 (FD40, dextran labeled with fluorescein isothiocyanate) 100 mg/L in the upper transwell chamber, filled the lower chamber with 600 *u*l PBS for 1hours, collected the PBS in the lower chamber, measured the intensity of FITC fluorescence with a fluorescence spectrometer, established a standard curve between fluorescence intensity and FD40 concentration, and used FD40 concentration to reflect the permeability of monolayer endothelial cells.

### 2.3. ELISA

HUVEC was seeded into a 96-well plate (1 × 10^5^/well). After stimulation with drugs as grouped, we took the supernatant and tested the concentration of MMP‐9 by ELISA method. The kit was purchased from Abcam (ab246539) and used following the kit instructions. The absorbance was measured at 450 nm with an ultraviolet spectrophotometer.

### 2.4. RT-PCR

After establishing the monolayer HUVEC model, we added drugs as grouped and extracted total RNA with TRIzol reagent for detection. We used ABI Prism 7500 system and 2X premixed SYBR Green (Enzynomics) to detect the mRNA abundance by real‐time quantitative PCR. The PCR conditions are as follows: 95°C (30 s) 1 cycle, and 95°C (5 s), 60°C (34 s), 40 cycles. The primers are as follows: HIF-1*α* upstream primer: 5′-CAGAGCAGGAAAGAGAGTCATAGAAC-3′ and HIF-1*α* downstream primer: 5′-TTTCGCTT CCTCTGAGCATTC-3′; MMP-9 upstream primer: 5′-GCC TGG CAC ATA GTA GGC CC-3′ and downstream primer: 5′-CTT CCT AGC CAG CCG GCA TC-3′; claudin-5 upstream primer: 5′-GAATTCGCCGCCACCATGGGGTCTGCAGCGTTG-3′ and downstream primer: 5′-GAATTCTCAGACATAGTTCTTCTTGTCGTAATCG-3′; ZO-1 upstream primer: 5′-CGA GTT GCAA TGG TTA ACG GA-3′ and downstream primer: 5′-TCA GGA TCA GGA CGA CTT ACT GG-3'; *β*-actin upstream primer: 5′-CCT GGC ACC CAG CAC AAT-3′ and downstream primer: 5′-GGG CGG GAC TCG TCA TAC-3′.

### 2.5. Western Blot

After establishing a monolayer HUVEC model, we added drugs as grouped and extracted total protein of the cells for detection.

An equal volume of 2× Laemmli sample buffer was added to the supernatant, and the supernatant mixture was boiled at 100°C for 5 minutes and stored at −20°C. Then, the centrifuge tube containing the supernatant mixture was thawed at 37°C. The same amount of protein and maker was loaded into the SDS-PAGE gel wells, with a sample amount of 20–30 *μ*g, and electrophoresis was imposed at 100 V for 1 to 2 hours. The antibody was purchased from Abcam, USA. The primary antibodies were rabbit antibodies, anti-MMP-9 antibody (ab76003), with a dilution ratio of 1 : 1000, anti-HIF-1*α* antibody (ab51608), with a dilution ratio of 1 : 500, anti-claudin 5 antibody (ab131259), with a dilution ratio of 1 : 5000, and the anti-ZO-1 antibody (ab276131), with a dilution ratio of 1 : 1000. The secondary antibodies were goat anti-rabbit antibodies (ab6721), with a dilution ratio of 1 : 3000. ImageJ software was used to quantify the gray value of the resulting graph and drew a statistical graph.

### 2.6. Immunocytochemistry

The immunofluorescence experiment was carried out according to the previously published method [19]. HUVEC was fixed with 4% paraformaldehyde for 30 minutes, washed twice with PBS, and then incubated with 1% hydrogen peroxide for 10 minutes. After two PBS washes, the cells were incubated with blocking solution (PBS containing 1% bovine serum albumin, 0.4% Triton X-100, and 4% normal goat serum) for 20 minutes. Next, the cells were incubated with primary antibodies, claudin-5 antibody (1 : 100, ab15106, Abcam), and ZO-1 antibody (1 : 500, ab221547, Abcam) at 4°C overnight. Then the cells were washed twice with PBS and labeled with fluorescein isothiocyanate (FITC) to culture secondary antibodies (1 : 500) for 1 hour at room temperature. The cover glass was fixed in a fixed medium, observed with a fluorescence microscope, and stained with DAPI (10 ug/ml).

### 2.7. Dual-Luciferase Reporter Experiment

By searching Ensemble and NCBI, two major gene databases, it was found that the human MMP-9 genome contains 7 hypoxia response elements 4000 bp before the transcription start site in the promoter region. According to the hypoxia response element position of the human MMP-9 promoter sequence, promoter fragments of different lengths were designed, and then the PCR primers of each promoter fragment were designed and synthesized. The whole blood DNA of healthy volunteers was extracted by the NaI method, human genomic DNA was used as a template, the promoter fragment was obtained by PCR amplification, the target DNA was cloned by T-A and transformed into *E. coli* Trans5*α*, and the plasmid was extracted for enzyme digestion identification. The identified target DNA fragments were connected to pGL3-Basic vector to construct a luciferase reporter plasmid, *E. coli* Trans5*α* was transformed, amplified, sequenced, and identified, the luciferase reporter plasmid already constructed was extracted, the plasmid was transfected into HUVEC, and the activity of the dual-luciferase reporter gene after TNF*α* stimulation was detected.

### 2.8. Statistical Analysis

All analyses involved in this study used unpaired *t*-tests to calculate *P* values. The result of *P* < 0.05 has statistical significance.

## 3. Results

### 3.1. Permeability Test of Monolayer Endothelial Cells

**Figure 1:**
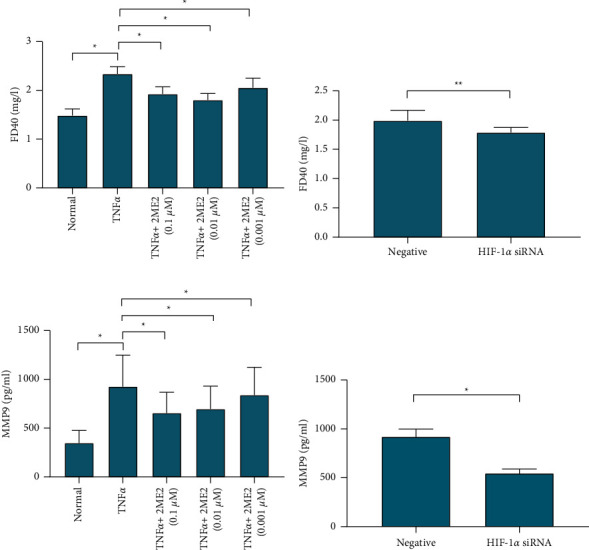
Permeability test of monolayer endothelial cells. (a) The effect of TNF*α* and TNF*α* combined with different concentrations of 2ME2 on the concentration of FD40. (b) The effect of HIF-1*α* siRNA transfection on the concentration of FD40. (c) Using ELISA method to detect the effect of TNF*α* and TNF*α* combined with different concentrations of 2ME2 on the concentration of MMP9 protein. ^*∗*^*P* < 0.05; ^*∗∗*^*P* < 0.01. (d) Using ELISA method to detect the effect of HIF‐1*α* siRNA transfection on the concentration of MMP9 protein.

In order to detect the permeability of human monolayer endothelial cells (HUVEC) under different treatment conditions, we tested the concentration of FD40 in different treatment groups. The concentration of FD40 in the lower chamber of the TNF*α* group was 2.339 ± 0.149 mg/l, which was significantly higher than that of the normal control group, which was 1.485 ± 0.137 mg/l. In the TNF*α* + 2ME2 group, after giving 0.1 uM, 0.01 uM, and 0.001 uM 2ME2 for coculture, the concentrations of FD40 were 1.927 ± 0.147, 1.803 ± 0.136, and 2.057 ± 0.193 mg/l, respectively. Compared with the TNF*α* group, *P* < 0.05, suggesting improved permeability (Figure 1(a)). The concentration of FD40 in the lower chamber of the HIF-1*α* siRNA-positive transfection group was 1.788 ± 0.088 mg/l, which was lower than 1.99 ± 0.176 mg/l (*P*=0.008) in the HIF-1*α* siRNA-negative control group, suggesting that after siRNA knocked down HIF-1*α* expression, the permeability decreased (Figure 1(b)).

In order to detect the effect of changes in cell permeability on the concentration of extracellular MMP9, we tested the concentration of MMP9 in the supernatant. The results showed that the concentration of MMP9 in the supernatant of the TNF*α* group was 926.6 ± 322.6 pg/ml, which was significantly higher than that of the normal control group of 348.2 ± 130.1 pg/ml. In the TNF*α* + 2ME2 group, after giving 0.1 uM, 0.01 uM, and 0.001 uM 2ME2, respectively, for coculture, the concentrations of MMP9 were 655.9 ± 213.66, 696.9 ± 235.27, and 839.5 ± 284.28 pg/ml, respectively, which were lower than those in the TNF*α* group, *P* < 0.05 (Figure 1(c)).

The concentration of MMP9 in the supernatant of the HIF-1*α* siRNA-positive transfection group was 545.9 ± 43.82 pg/ml, which was lower than the HIF-1*α*siRNA-negative control group 921.1 ± 76.48 pg/ml (*P*=0.03), indicating that after siRNA knocks down the expression of HIF-1*α*, the concentration of MMP9 in the supernatant is reduced (Figure 1(d)).

### 3.2. HIF-1*α*, MMP-9, Claudin-5, and ZO-1 DNA Expression Changes

**Figure 2:**
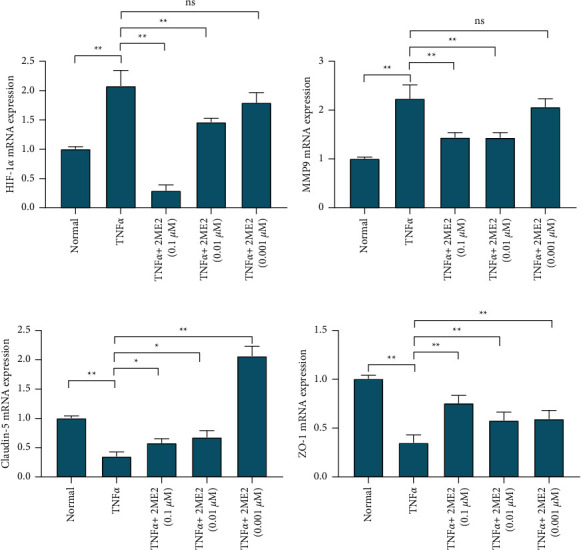
Changes in DNA levels of HIF-1*α*, MMP-9, claudin-5, and ZO-1 RT-PCR detected the effect of TNF*α* and TNF*α* combined with different concentrations of 2ME2 on the expression of HIF-1*α* (a), MMP-9 (b), claudin-5 (c), and ZO-1 (d) DNA levels. ^*∗*^*P* < 0.05; ^*∗∗*^*P* < 0.01; ns, no significant difference (no significance).

The relative expression of HIF-1*α* mRNA in the normal control group was 1.006 ± 0.036, and the relative expression in the TNF*α* group was 2.080 ± 0.267, which was significantly higher than that in the normal control group, *P* < 0.01; in the TNF *α* + 2ME2 0.1 uM group, the TNF*α* + 2ME2 0.01 uM group, and the TNF*α* + 2ME2 0.001 uM group, the relative expression of HIF-1*α* mRNA was 0.292 ± 0.099, 1.464 ± 0.066, and 1.795 ± 0.172, respectively. 0.1 uM and 0.01 uM 2ME2 can inhibit the high expression of HIF-1*α* caused by TNF*α*, *P* < 0.01. The inhibitory effect disappeared with 0.001 uM 2ME2, *P*=0.145 (Figure 2(a)).

The relative expression of MMP9 mRNA in the normal control group was 1.006 ± 0.036, and the relative expression in the TNF*α* group was 2.237 ± 0.279, which was significantly higher than that in the normal control group, *P* < 0.01; in the TNF*α* + 2ME2 0.1 uM group, the TNF*α* + 2ME2 0.01 uM group, and the TNF*α* + 2ME2 0.001 uM group, the relative expression of HIF-1*α* mRNA was 1.440 ± 0.101, 1.437 ± 0.103, and 2.063 ± 0.170, respectively. 0.1 uM and 0.01 uM 2ME2 can inhibit the high expression of HIF-1*α* caused by TNF*α*, *P* < 0.01. The inhibitory effect was not significant with 0.001 uM 2ME2, *P*=0.686 (Figure 2(b)).

The relative expression of claudin-5 mRNA in the normal control group was 1.006 ± 0.036, and the relative expression of claudin-5 mRNA in the TNF*α* group was 0.350 ± 0.080, which was lower than that in the normal control group, *P* < 0.01; after adding 2ME2 for intervention, in the TNF*α* + 2ME2 0.1 uM group, the TNF*α* + 2ME2 0.01 uM group, and the TNF*α* + 2ME2 0.001 uM group, the relative expression of claudin-5mRNA was 0.580 ± 0.084, 0.669 ± 0.073, and 0.679 ± 0.113, respectively. Compared with the TNF*α* group, the expression of claudin-5 mRNA was significantly increased, *P* < 0.01 (Figure 2(c)).

The relative expression of ZO-1 mRNA in the normal control group was 1.006 ± 0.036, and the relative expression of ZO-1 mRNA in the TNF*α* group was 0.350 ± 0.080, which was lower than that in the normal control group, *P* < 0.01; after adding 2ME2 for intervention, in the TNF*α* + 2ME2 0.1 uM group, the TNF*α* + 2ME2 0.01 uM group, and the TNF*α* + 2ME2 0.001 uM group, the relative expression of ZO-1 mRNA was 0.756 ± 0.079, 0.580 ± 0.086, and 0.594 ± 0.088, respectively. Compared with the TNF*α* group, the expression of ZO-1 was significantly increased, *P* < 0.01 (Figure 2(d)).

**Figure 3:**
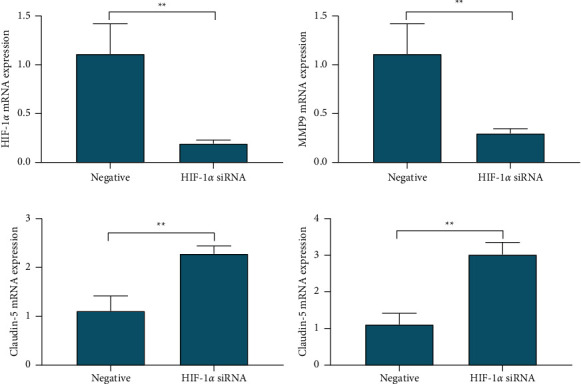
HIF-1*α*, MMP-9, claudin-5, and ZO-1 DNA expression changes. RT-PCR was used to detect the effect of HIF-1*α* siRNA transfection on the level of DNA expression of HIF-1*α* (a), MMP-9 (b), claudin-5 (c), and ZO-1 (d). ^*∗∗*^*P* < 0.01.

The relative expression of HIF-1*α* and MMP-9 mRNA in the HIF-1*α* siRNA-positive transfection group was 0.193 ± 0.036 and 0.298 ± 0.047, respectively, which was lower than 1.112 ± 0.307 in the HIF-1*α* siRNA-negative control group, *P* < 0.01; the relative expression of claudin-5 and ZO-1mRNA was 2.282 ± 0.158 and 3.021 ± 0.3259, respectively, which was higher than 1.112 ± 0.307 in the HIF-1*α* siRNA-negative control group, *P* < 0.01. After siRNA interfered with the expression of HIF-1*α*, the expression of MMP-9 mRNA decreased accordingly, and the expression of claudin-5 and ZO-1 mRNA increased (Figure 3).

### 3.3. HIF-1*α*, MMP-9, Claudin-5, and ZO-1 Protein Expression Changes

The relative expression of HIF-1*α*/*β* actin protein in the normal control group was 0.184 ± 0.016, and the relative expression of HIF-1*α*/*β* actin protein in the TNF*α* group was 0.403 ± 0.015, which was significantly higher than that in the normal control group, *P* < 0.01; 2ME2 can inhibit HIF-1*α* expression. The relative expression of HIF-1*α*/*β* actin protein in the TNF*α* + 2ME2 0.1 uM group, the TNF*α* + 2ME2 0.01 uM group, and the TNF*α* + 2ME2 0.001 uM group was 0.282 ± 0.015, 0.318 ± 0.018, and 0.413 ± 0.030, respectively. 0.1 uM and 0.01 uM 2ME2 can inhibit the high expression of HIF-1*α* caused by TNF*α*, *P* < 0.01. The inhibitory effect disappeared with 0.001uM 2ME2, *P*=0.288.

**Figure 4:**
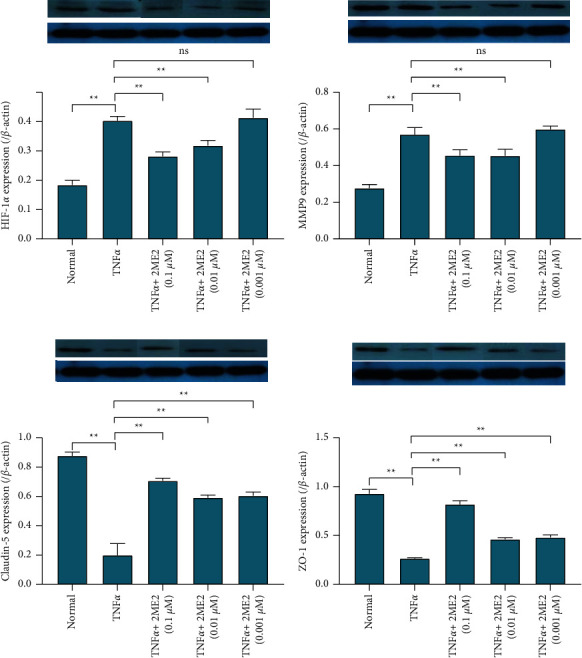
HIF-1*α*, MMP-9, claudin-5, and ZO-1 protein expression changes. Western blot was used to detect the effects of TNF*α* and TNF*α* combined with different concentrations of 2ME2 on the protein expression of HIF-1*α* (a), MMP-9 (b), claudin-5 (c), and ZO-1 (d). ^*∗∗*^*P* < 0.01, ns (no significance).

The relative expression of MMP-9/*β* actin protein in the normal control group was 0.276 ± 0.021, and the relative expression of MMP-9/*β* actin protein in the TNF*α* group was 0.570 ± 0.038, which was higher than that in the normal control group, *P* < 0.01; after adding 2ME2 for intervention, the relative expression of MMP-9/*β* actin protein in the TNF*α* + 2ME2 0.1 uM group, the TNF*α* + 2ME2 0.01 uM group, and the TNF*α* + 2ME2 0.001 uM group was 0.455 ± 0.032, 0.454 ± 0.036, and 0.598 ± 0.018, respectively (*P* < 0.01, *P* < 0.01, and *P*=0.288). Compared with the TNF*α* group, the expression of MMP-9 protein in the TNF*α* + 2ME2 0.01 uM group and the TNF*α* + 2ME2 0.001 uM group was significantly reduced, and the expression of MMP-9 protein in the TNF*α* + 0.001 uM 2ME2 group was slightly increased, *P*=0.408 (Figure 4(a)).

The relative expression of claudin-5/*β* actin protein in the normal control group was 0.877 ± 0.026, and the relative expression of claudin-5/*β* actin protein in the TNF*α* group was 0.199 ± 0.079, which was lower than that in the normal control group, *P* < 0.01; after adding 2ME2 for intervention, the relative expression of claudin-5/*β* actin protein in the TNF*α* + 2ME2 0.1 uM group, the TNF*α* + 2ME2 0.01 uM group, and the TNF*α* + 2ME2 0.001 uM group was 0.707 ± 0.018, 0.591 ± 0.019, and 0.605 ± 0.025, respectively. Compared with the TNF*α* group, the expression of claudin-5 was significantly increased, *P* < 0.01 (Figure 4(b)).

The relative expression of ZO-1/*β* actin protein in the normal control group was 0.928 ± 0.045, and the relative expression of ZO-1/*β* actin protein in the TNF*α* group was 0.264 ± 0.009, which was lower than that in the normal control group, *P* < 0.01; after adding 2ME2 for intervention, the relative expression of ZO-1/*β* actin protein in the TNF*α* + 2ME2 0.1 uM group, the TNF*α* + 2ME2 0.01 uM group, and the TNF*α* + 2ME2 0.001 uM group was 0.819 ± 0.037, 0.462 ± 0.015, and 0.481 ± 0.026, respectively. Compared with the TNF*α* group, the expression of ZO-1 protein was increased, *P* < 0.01 (Figure 4(c)).

**Figure 5:**
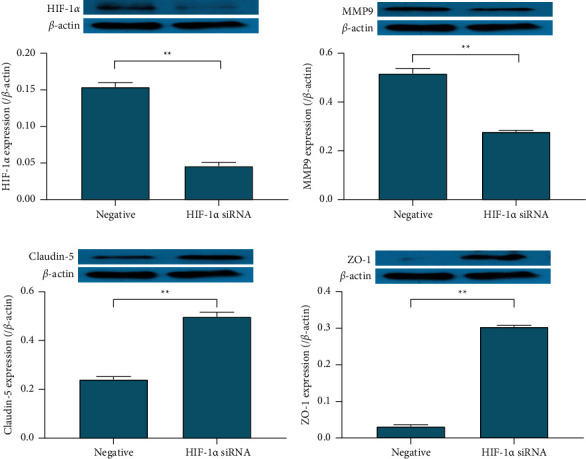
HIF1*α*, MMP-9, claudin-5, and ZO-1 protein expression changes. Western blot was used to detect the effect of HIF-1*α* siRNA transfection on the protein expression of HIF-1*α* (a), MMP-9 (b), claudin-5 (c), and ZO-1 (d). ^*∗∗*^*P* < 0.01.

The relative expression of HIF-1*α*/*β* actin protein in the HIF-1*α* siRNA-positive transfection group was 0.046 ± 0.005, which was lower than 0.154 ± 0.006 in the HIF-1*α* siRNA-negative control group (*P* < 0.01), suggesting that after siRNA knocked down the HIF-1*α* expression, the HIF-1*α* expression caused by TNF*α* interfered. The relative expression of MMP-9/*β* actin protein in the HIF-1*α* siRNA-positive transfection group was 0.278 ± 0.006, which was lower than 0.517 ± 0.021 in the HIF-1*α* siRNA-negative control group (*P* < 0.01), indicating that after siRNA interfered with the HIF-1*α* expression, the MMP-9 high expression was inhibited. The relative expression of claudin-5/*β* actin protein in the HIF-1*α* siRNA-positive transfection group was 0.498 ± 0.018, which was lower than 0.241 ± 0.011 in the HIF-1*α* siRNA-negative control group (*P* < 0.01), suggesting that after siRNA interfered with the HIF-1*α* expression, the expression of claudin-5 was promoted. The relative expression of ZO-1/*β* actin protein in the HIF-1*α* siRNA-positive transfection group was 0.304 ± 0.004, which was lower than 0.032 ± 0.004 in the HIF-1*α* siRNA-negative control group (*P* < 0.01), suggesting that after siRNA interfered with the HIF-1*α* expression, the expression of ZO-1 was promoted (Figure 5).

### 3.4. Immunofluorescence Detection of the Effect of TNF*α* on the Expression of Claudin-5 and ZO-1 in Human Umbilical Vein Endothelial Cells

**Figure 6:**
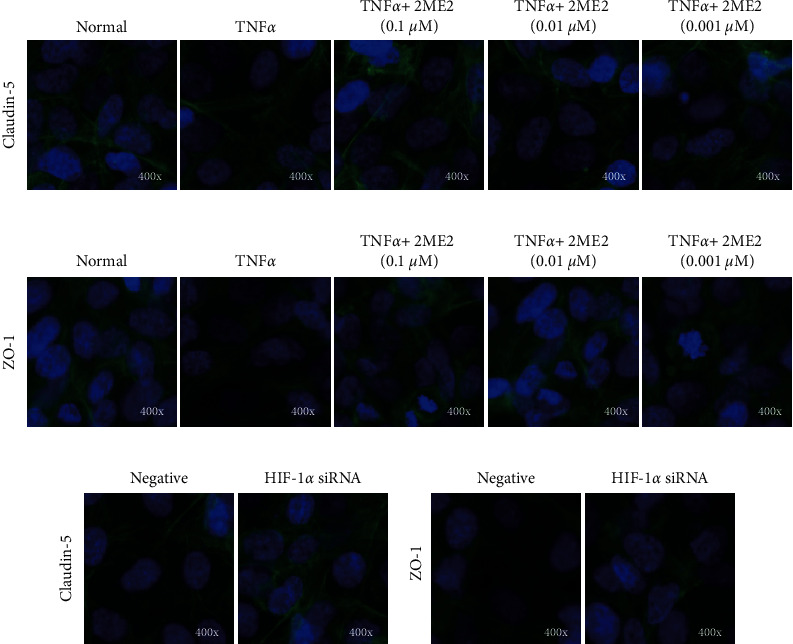
Immunofluorescence detection of the effect of TNF*α* on the expression of claudin-5 and ZO-1 in human umbilical vein endothelial cells. (a) and (c) The effect of TNF*α*, TNF*α* combined with 2ME2 and siRNA transfection on the expression of claudin-5. (b, d) The effect of TNF*α*, TNF*α* combined with 2ME2 and siRNA transfection on the expression of ZO-1.

The fluorescent pattern of claudin-5 and ZO-1 proteins in the normal control group was continuous, and there were many fluorescent particles. The fluorescent pattern of claudin-5 and ZO-1 proteins in the TNF*α* group showed an obvious break, and the fluorescent particles were significantly reduced. The fluorescence pattern of claudin-5 and ZO-1 protein was more continuous than that of the TNF*α* group, with more fluorescent particles (Figure 6(a)). The fluorescent pattern of claudin-5 and ZO-1 proteins in the HIF-1*α* siRNA-positive transfection group was more continuous than that of the HIF-1*α* siRNA-negative control group, and the number of fluorescent particles increased (Figure 6(b)).

### 3.5. Mechanism of HIF-1*α*′s Direct Initiation of MMP-9 at the Transcription Level

**Figure 7:**
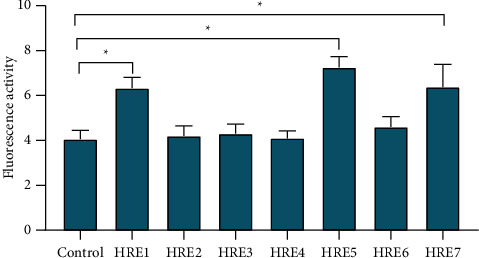
Dual-luciferase experiment to explore the mechanism of HIF-1*α*′s direct activation of MMP-9 at the transcription level.

In order to explore the mechanism of HIF-1*α*′s direct activation of MMP-9 at the transcription level, we searched Ensemble and NCBI, two major gene databases, and found that the human MMP-9 genome has 7 hypoxia response elements in a length of 4000 bp before the transcription start site in the promoter region. Through the double luciferase reporter gene experiment, it was found that the activities of HRE1, HRE5, and HRE7 after TNF*α* stimulation were significantly higher than those of other HREs (6.33 ± 0.482, 7.25 ± 0.488, and 6.38 ± 1.010, *P* < 0.05, Figure 7).

## 4. Discussion

**Figure 8:**
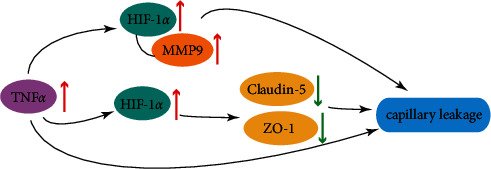
Mechanism diagram.

This study found that, under the action of TNF*α*, the permeability of the HUVEC monolayer increased and the concentration of MMP-9 in the cell supernatant increased (PMID: 30976100). 2ME2 and HIF-1*α*-siRNA transfection can improve the above situation (*P* < 0.05). TNF*α* is a proinflammatory factor secreted by macrophages and endothelial cells, which is related to the increase of plaque load [20]. Recent literature reported that TNF*α* can lead to the loss of adhesion protein, thereby increasing cell permeability [21]. In our results, the extracellular FD40 concentration under the action of TNF*α* increased significantly, indicating an increase in cell permeability, which is consistent with previous reports. However, with the addition of the HIF-1*α* inhibitor 2-methoxyestradiol (2ME2), the increasing trend of FD40 concentration was inhibited, and with the increase of the 2ME2 concentration, the inhibitory effect gradually increased. Besides, the addition of HIF-1*α* siRNA would also significantly decrease the concentration of FD40, suggesting that TNF*α* may affect cell permeability by regulating HIF-1*α* (Figure 8).

By investigating the literature, we found that HIF-1*α* can affect the expression of MMP-9 [22, 23]. Therefore, we hypothesized the influence mechanism of HIF-1*α* on cell permeability. Perhaps HIF-1*α* affects cell permeability by affecting the expression of MMP9. We tested the expression of MMP9 through ELISA experiments. The results showed that TNF*α* can induce the expression of MMP9, while the addition of 2ME2 and HIF-1*α*-siRNA can slow down this process, which confirmed our conjecture from the side. Subsequently, we further verified our conjecture through RT-PCR and Western blot experiments. The results showed that TNF*α* can induce the expression of HIF-1*α* at the mRNA level and protein level, which is consistent with the report by Tsapournioti et al. [24]. 2ME2 and HIF-1*α*-siRNA transfection can inhibit the expression of HIF-1*α* caused by TNF*α*. At the same time, TNF*α* can also induce the expression of MMP9 at the mRNA level and protein level, which is also consistent with previous reports [25]. The addition of 2ME2 and HIF-1*α*-siRNA can also inhibit the ability of TNF*α* to regulate MMP9, which has once again confirmed the regulatory effect of HIF-1*α* on MMP9.

In addition, in order to further explore the correlation between TNF*α* and HIF-1*α* and cell permeability, we also tested the expression of the membrane protein claudin-5 and tight junction protein ZO-1. Claudin-5 is a member of the intact membrane protein claudins and a key component of the TJ chain, especially in brain endothelial cells [19]. It has been proved to have mediated changes in endothelial or epithelial permeability in many pathological diseases, including ischemia [26]. In our research results, TNF*α* can increase the expression of claudin-5, while 2ME2 and HIF-1*α*-siRNA can inhibit this process. ZO-1 is involved in cell adhesion and tight junction. As an important structural protein of tight junction, ZO-1 maintains the integrity of the tight junction complex mainly by connecting claudins, occlusion proteins, and cytoskeleton proteins. Abnormal expression of ZO-1 may hinder the formation of tight junction between cells [27, 28]. In our research results, the expression trend of ZO-1 is consistent with claudin-5. This once again indicates from the side that TNF*α* and HIF-1*α* will affect the permeability of cell membranes.

In order to have a more intuitive understanding of the regulatory relationship between HIF-1*α* and MMP9, we searched the database, designed primers, and used dual-luciferase experiments to explore the possible sites. Finally, it was found that element 1, element 5, and element 7 of HIF-1*α* were the sites that interact with MMP-9 at the transcription level, confirming the interaction between HIF-1*α* and MMP9.

## 5. Conclusions

All in all, this study showed that, under the action of TNF*α*, the permeability of monolayer endothelial cells increased, and the concentration of MMP-9 in the cell supernatant increased. 2ME2 and HIF-1*α*-siRNA transfection can improve the above situation (*P* < 0.05). 2ME2 and HIF-1*α*-siRNA transfection can inhibit the high expression of HIF-1*α* and MMP-9 caused by TNF*α*, thereby increasing the expression of claudin-5 and ZO-1 (*P* < 0.05). 2ME2 and HIF-1*α*-siRNA transfection can improve the inhibition of TNF*α* on the expression of cell membrane protein claudin-5 and tight junction protein ZO-1. Element 1 (−3798 bp), element 5 (−1878 bp), and element 7 (−1489 bp) were the sites where HIF-1*α* interacted with MMP-9 at the transcription level.

## Data Availability

The data used to support the findings of this study are available from the corresponding author upon request.

## References

[1] Montesi S. B., Rao R., Liang L. L. (2018). Gadofosveset-enhanced lung magnetic resonance imaging to detect ongoing vascular leak in pulmonary fibrosis. *European Respiratory Journal*.

[2] Amado-Azevedo J., van Stalborch A. D., Valent E. T. (2021). Depletion of Arg/Abl2 improves endothelial cell adhesion and prevents vascular leak during inflammation. *Angiogenesis*.

[3] Goldenberg N. M., Steinberg B. E., Slutsky A. S., Lee W. L. (2011). Broken barriers: a new take on sepsis pathogenesis. *Science Translational Medicine*.

[4] Teuwen L.-A., Geldhof V., Pasut A., Carmeliet P. (2020). COVID-19: the vasculature unleashed. *Nature Reviews Immunology*.

[5] Matthay M. A., Ware L. B., Zimmerman G. A. (2012). The acute respiratory distress syndrome. *Journal of Clinical Investigation*.

[6] Zheng H., Yu S., Zhu C., Guo T., Liu F., Xu Y. (2021). HIF1*α* promotes tumor chemoresistance via recruiting GDF15-producing TAMs in colorectal cancer. *Experimental Cell Research*.

[7] Yu L., Shu H., Xing L. (2020). Silencing long noncoding RNA NEAT1 suppresses the tumorigenesis of infantile hemangioma by competitively binding miR33a5p to stimulate HIF1alpha/NFkappaB pathway. *Molecular Medicine Reports*.

[8] Wang C., Luo J., Chen Z. (2021). MiR-375 impairs the invasive capabilities of hepatoma cells by targeting HIF1*α* under hypoxia. *Digestive Diseases and Sciences*.

[9] Zhu W. J., Chang B. Y., Wang X. F. (2020). FBW7 regulates HIF-1alpha/VEGF pathway in the IL-1beta induced chondrocytes degeneration. *European Review for Medical and Pharmacological Sciences*.

[10] Zhang J., He J., Luo Y., Liu Y., Fan X. (2021). miR-210 regulates the inflammation of otitis media with effusion by inhibiting the expression of hypoxia-inducible factor (HIF)-1a. *Biochemical and Biophysical Research Communications*.

[11] Mas-Bargues C., Sanz-Ros J., Roman-Dominguez A. (2020). Extracellular vesicles from healthy cells improves cell function and stemness in premature senescent stem cells by miR-302b and HIF-1alpha activation. *Biomolecules*.

[12] Sioustis I. A., Martu M. A., Aminov L. (2021). Salivary metalloproteinase-8 and metalloproteinase-9 evaluation in patients undergoing fixed orthodontic treatment before and after periodontal therapy. *International Journal of Environmental Research and Public Health*.

[13] Matusiak M., OzieRblo D., Obrycka A. (2021). Functional polymorphism of MMP9 and BDNF as potential biomarker of auditory neuroplasticity in prelingual deafness treatment with cochlear implantation-A retrospective cohort analysis. *Trends in Hearing*.

[14] Rodriguez-Sanchez E., Navarro-Garcia J. A., Aceves-Ripoll J. (2020). Variations in circulating active MMP-9 levels during renal replacement therapy. *Biomolecules*.

[15] Peltonen R., Hagström J., Tervahartiala T., Sorsa T., Haglund C., Isoniemi H. (2021). High expression of MMP-9 in primary tumors and high preoperative MPO in serum predict improved prognosis in colorectal cancer with operable liver metastases. *Oncology*.

[16] Zhou L., Ren M., Zeng T. (2019). TET2-interacting long noncoding RNA promotes active DNA demethylation of the MMP-9 promoter in diabetic wound healing. *Cell Death and Disease*.

[17] Ulrich C. C., Arinze V., Wandscheer C. B. (2019). Matrix metalloproteinases 2 and 9 are elevated in human preterm laboring uterine myometrium and exacerbate uterine contractility†. *Biology of Reproduction*.

[18] Hui L., Nie Y., Li S. (2020). Matrix metalloproteinase 9 facilitates Zika virus invasion of the testis by modulating the integrity of the blood-testis barrier. *PLoS Pathogens*.

[19] Lv J.-M., Guo X.-M., Chen B., Lei Q., Pan Y.-J., Yang Q. (2016). The noncompetitive AMPAR antagonist perampanel abrogates brain endothelial cell permeability in response to ischemia: involvement of claudin-5. *Cellular and Molecular Neurobiology*.

[20] Battes L. C., Cheng J. M., Oemrawsingh R. M. (2014). Circulating cytokines in relation to the extent and composition of coronary atherosclerosis: results from the ATHEROREMO-IVUS study. *Atherosclerosis*.

[21] Hardyman M. A., Wilkinson E., Martin E. (2013). TNF-*α*-mediated bronchial barrier disruption and regulation by src-family kinase activation. *The Journal of Allergy and Clinical Immunology*.

[22] Li P., Butcher N. J., Minchin R. F. (2019). Arylamine N-acetyltransferase 1 regulates expression of matrix metalloproteinase 9 in breast cancer cells: role of hypoxia-inducible factor 1-*α*. *Molecular Pharmacology*.

[23] Meng L., Cheng Y., Tong X. (2018). Tumor oxygenation and hypoxia inducible factor-1 functional inhibition via a reactive oxygen species responsive nanoplatform for enhancing radiation therapy and abscopal effects. *ACS Nano*.

[24] Tsapournioti S., Mylonis I., Hatziefthimiou A. (2013). TNF*α* induces expression of HIF-1*α* mRNA and protein but inhibits hypoxic stimulation of HIF-1 transcriptional activity in airway smooth muscle cells. *Journal of Cellular Physiology*.

[25] Woodward A. M., Di Zazzo A., Bonini S., Argüeso P. (2020). Endoplasmic reticulum stress promotes inflammation-mediated proteolytic activity at the ocular surface. *Scientific Reports*.

[26] Jia W., Lu R., Martin T. A., Jiang W. G. (2014). The role of claudin-5 in blood-brain barrier (BBB) and brain metastases (review). *Molecular Medicine Reports*.

[27] Xu J., Liang R., Zhang W. (2020). Faecalibacterium prausnitzii ‐derived microbial anti‐inflammatory molecule regulates intestinal integrity in diabetes mellitus mice via modulating tight junction protein expression. *Journal of Diabetes*.

[28] König J., Wells J., Cani P. D. (2016). Human intestinal barrier function in health and disease. *Clinical and Translational Gastroenterology*.

